# Long-Read Genome Sequencing and Assembly of *Leptopilina boulardi*: A Specialist *Drosophila* Parasitoid

**DOI:** 10.1534/g3.120.401151

**Published:** 2020-03-26

**Authors:** Shagufta Khan, Divya Tej Sowpati, Arumugam Srinivasan, Mamilla Soujanya, Rakesh K. Mishra

**Affiliations:** CSIR – Centre for Cellular and Molecular Biology, Hyderabad – 500007, Telangana, India

**Keywords:** *Leptopilina boulardi*, wasp, parasitoid, *Drosophila*, genome assembly

## Abstract

*Leptopilina* *boulardi* (Hymenoptera: Figitidae) is a specialist parasitoid of *Drosophila*. The *Drosophila-Leptopilina* system has emerged as a suitable model for understanding several aspects of host-parasitoid biology. However, a good quality genome of the wasp counterpart was lacking. Here, we report a whole-genome assembly of *L. boulardi* to bring it in the scope of the applied and fundamental research on *Drosophila* parasitoids with access to epigenomics and genome editing tools. The 375Mb draft genome has an N50 of 275Kb with 6315 scaffolds >500bp and encompasses >95% complete BUSCOs. Using a combination of *ab-initio* and RNA-Seq based methods, 25259 protein-coding genes were predicted and 90% (22729) of them could be annotated with at least one function. We demonstrate the quality of the assembled genome by recapitulating the phylogenetic relationship of *L. boulardi* with other Hymenopterans. The key developmental regulators like Hox genes and sex determination genes are well conserved in *L. boulardi*, and so is the basic toolkit for epigenetic regulation. The search for epigenetic regulators has also revealed that *L. boulardi* genome possesses DNMT1 (maintenance DNA methyltransferase), DNMT2 (tRNA methyltransferase) but lacks the *de novo* DNA methyltransferase (DNMT3). Also, the heterochromatin protein 1 family appears to have expanded as compared to other hymenopterans. The draft genome of *L. boulardi* (Lb17) will expedite the research on *Drosophila* parasitoids. This genome resource and early indication of epigenetic aspects in its specialization make it an interesting system to address a variety of questions on host-parasitoid biology.

Parasitoids are organisms that have a non-mutualistic association with their hosts (Eggleton and Belshaw 1992; Godfray 1994). Around 10–20% of the described insect species are estimated to be parasitoids. They are spread across five insect orders, *i.e.*, Hymenoptera, Diptera, Coleoptera, Lepidoptera, Neuroptera, Strepsiptera and Trichoptera (Eggleton and Belshaw 1992; LaSalle and Gauld 1991; Heraty 2009), among which the vast majority are parasitoid wasps belonging to the order Hymenoptera (LaSalle and Gauld 1991; Godfray 1994; Kraaijeveld *et al.* 1998). Depending on the stage of the host they attack, they are categorized into the egg, larval, pupal or adult parasitoids. The larvae of parasitoids either feed/develop within the host without impeding its growth (endoparasitic koinobionts) or live on the host after killing or permanently paralyzing it (ectoparasitic idiobionts) (Godfray 1994; Kraaijeveld*et al*. 1998). Based on the host preference, parasitoids are further classified as generalists and specialists: generalists can parasitize a broad range of species, whereas specialists favor one or two host species (Lee *et al.* 2009). Likewise, hymenopteran parasitoids display a repertoire of unique features such as polyembryony, hyper-/mutli-/superparasitism, complex multi-level interactions, and haplodiploid sex-determination (Godfray 1994). Many studies have also demonstrated their potential in the biological control of insect pests (Heraty 2009; Figueiredo *et al.* 2015; Smith 1996; Martínez, González, and Dicke 2018; Machtinger *et al.* 2015; Kruitwagen, Beukeboom, and Wertheim 2018).

*Leptopilina boulardi* (NCBI taxonomy ID: 63433) is a solitary parasitoid wasp from the Figitidae family in the Hymenoptera order (Figure 1). It is a cosmopolitan species, which is ubiquitously found in the Mediterranean and intertropical environments. *L. boulardi* parasitizes *Drosophila melanogaster* and *Drosophila simulans* at second- to early third-instar larval stages and hence, is a specialist (Fleury *et al.* 2009). However, few strains of the wasp can also infect other Drosophilids like *D. yakuba*, *D. subobscura* and *D. pseudoobscura*, albeit to a lesser extent (Dubuffet *et al.* 2008; Schlenke *et al.* 2007). *Leptopilina*, like all the other Hymenopterans, has a haplodiploid sex-determination system. The females are diploid and males are haploid. They are endoparasitic koinobionts, *i.e.*, they lay eggs inside the host larva, allowing the host to grow and feed without rapidly killing it (Fleury *et al.* 2009; Lee *et al.* 2009; Kaiser, Couty, and Perez-Maluf 2009). During oviposition, the parasitoid co-inject virulence factors like venom proteins, Virus-like Particles (VLPs) into the larval hemolymph, which helps in taming the host immune responses (Dupas *et al.* 1996; Goecks *et al.* 2013; Gueguen *et al.* 2011). After hatching inside the host hemocoel, the parasitoid larva histolyzes the host tissues gradually. Subsequently, the endoparasitoid transitions into an ectoparasitoid and consumes the host entirely while residing inside the host puparium until emergence. The entire life cycle takes 21-22 days at 25° (Kaiser, Couty, and Perez-Maluf 2009; Fleury *et al.* 2009). Alternatively, the host can mount an immune response leading to the death of the parasitoid by encapsulation and the emergence of the host. The interaction, to some degree, also culminates in the death of both host and parasitoid (Fleury *et al.* 2009; Rizki and Rizki 1990; Small *et al.* 2012; A R Kraaijeveld *et al.* 1998). Such paradigms of evolutionary arms-race are prevalent in insects. However, the combination of *Drosophila* and *Leptopilina*, in particular, has unfolded as a promising tool to study various aspects of the host-parasitoid biology such as coevolutionary dynamics, behavioral ecology, physiology, innate-immune responses, superparasitism (Lee *et al.* 2009; Fellowes and Godfray 2000; Kraaijeveld *et al.* 2009; Tracy Reynolds and Hardy 2004; Kraaijeveld *et al.* 1998). The advancement could also be attributed to the well-established and extensively studied host.

**Figure 1:**
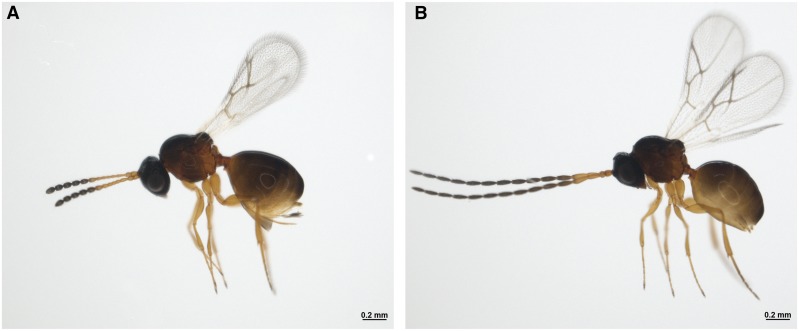
Bright field image of *Leptopilina boulardi* (Lb17 strain). A) Adult female and B) adult male.

The genotype, age, size, and nutritional conditions of the host affect the success of the parasitoid (Boulétreau and Wajnberg 1986; Godfray 1994). Nevertheless, the virulence of the parasitoid is a key factor in determining the fate of an infected host. Although, studies have explored the cause of genetic variance (intra- or inter-specific) and identified the genes involved in the host resistance (Kraaijeveld *et al.* 1998; Hita *et al.* 1999; Schlenke *et al.* 2007; Howell *et al.* 2012; Salazar-Jaramillo *et al.* 2017), our understanding of the genetic and epigenetic basis of variation seen in the counter-resistance/virulence of the parasitoids is limited (Kraaijeveld *et al.* 2009; Colinet *et al.* 2010). Another factor that affects the outcome of the host-parasitoid association is the symbiotic partners they harbor, such as *Leptopilina boulardi* Filamentous Virus (LbFV) and *Wolbachia*. LbFV, specific to *L. boulardi*, causes the females to lay eggs in an already parasitized host (superparasitism). Thereby favoring its transmission, and indirectly helping the parasitoid dodge the immune system of the host (Julien Varaldi *et al.* 2003, 2009; Lepetit *et al.* 2017; J. Varaldi and Lepetit 2018; Martinez *et al.* 2012; Patot *et al.* 2012, 2009). *Wolbachia*, an alpha-proteobacterium, is also the most prevalent endosymbiont of Arthropods. It manipulates the reproductive machinery of the host by inducing either of the following: feminization, male-specific killing, parthenogenesis and cytoplasmic incompatibility, and enhance their transmission to the subsequent generation (Werren, Windsor, and Guo 1995; Vavre, Mouton, and Pannebakker 2009). Hymenopterans, with haplodiploid sex determination, are appropriate hosts for parthenogenesis-inducing *Wolbachia* and have been implicated in the evolution of asexual lineages, such as in *L. clavipes* (K. Kraaijeveld *et al.* 2016). Interestingly, these bacterial parasites fail to infect the Boulardi clade of the *Leptopilina* genus, unlike the Heterotoma and Clavipes clades (Vavre, Mouton, and Pannebakker 2009; Heath *et al.* 1999). Such dichotomy observed in the susceptibility of *Drosophila* parasitoids to infections remains elusive.

The epigenetic mechanisms underlying such multispecies interactions that result in the manipulation of behavior and life-history traits of *Leptopilina* genus have not been investigated yet. Therefore, knowing the genomes of *Drosophila* parasitoids will be of great significance in providing a detailed insight into their biology. In this study, we have sequenced the whole genome of *Leptopilina boulardi* (Lb17), generated a high-quality genome assembly, and annotated it. We further looked for its phylogenetic relationship with select metazoans, conservation of genes responsible for body patterning, sex determination and epigenetic regulation of gene expression.

## Materials And Methods

### Sample collection

*L. boulardi* (Lb17 strain), kindly provided by S. Govind (Biology Department, The City College of the City University of New York), was reared on *D. melanogaster* (Canton-S strain) as described earlier (Small *et al.* 2012). Briefly, 50-60 young flies were allowed to lay eggs for 24 hr at 25° in vials containing standard yeast/corn-flour/sugar/agar medium. Subsequently, the host larvae were exposed to six to eight male and female wasps, respectively, 48 hr after the initiation of egg lay. The culture conditions were maintained at 25° and LD 12:12. The wasps (two days old) were collected, flash-frozen in liquid nitrogen, and stored at -80° until further use.

### Genomic DNA preparation and sequencing

For whole-genome sequencing on Illumina HiSeq 2500 platform (Table 1), the genomic DNA was extracted as follows: 100 mg of wasps were ground into a fine powder in liquid nitrogen and kept for lysis at 55° in SNET buffer overnight (400 mM NaCl, 1% SDS, 20 mM Tris-HCl pH 8.0, 5 mM EDTA pH 8.0 and 2 mg/ml Proteinase K) with gentle rotation at 10 rpm. Next day, after RNase A (100 μg/ml) digestion, Phenol: Chloroform: Isoamyl Alcohol extraction was performed, followed by Ethanol precipitation. The pellet was resuspended in 1X Tris-EDTA buffer (pH 8.0).

**Table 1:** SUMMARY STATISTICS OF THE ASSEMBLED GENOME

Genome assembly	Numbers
Assembly size 1n (bp)	375,731,061
Number of N’s (before gapfilling)	1,423,533
Number of N’s (after gapfilling)	1,216,865
GC content (%)	28.26
Number of scaffolds	6315
N50 (bp)	275,616
Largest scaffold (bp)	2,405,804
Average scaffold size (bp)	59,254

For long-read sequencing on PacBio Sequel II platform, the genomic DNA preparation was done from 200 mg wasps using the protocol described earlier (Mayjonade *et al.* 2016) with the following additional steps: Proteinase K digestion for 30 min at 50° after lysis, RNase A digestion for 10-15 min at RT (1 μl per 100 μl of 100 mg/ml) after the centrifugation step of contaminant precipitation with potassium acetate and a single round of Phenol: Chloroform: Isoamylalcohol (25:24:1, v/v) (Cat. No. 15593031) phase separation before genomic DNA purification using Agencourts AMPure XP beads (Item No. A63880).

### Hybrid genome assembly and assessment of genome completeness

Assembly of the reads was done using a hybrid assembler, MaSuRCA (Zimin *et al.* 2013). GapFiller (Nadalin, Vezzi, and Policriti 2012) was used to fill N’s in the assembly. Following gap filling, all scaffolds shorter than 500bp were removed from the assembly. The version thus obtained was used for all further analyses. For assessing the quality of the genome assembly, bowtie2 (Langmead and Salzberg 2012) and BUSCOv3 (Simão *et al.* 2015) was used.

### Identification of repeat elements

To identify repeat elements in the *L. boulardi* assembly, RepeatModeler was used with RepeatScout (Price, Jones, and Pevzner 2005) and TRF (Benson 1999) to generate a custom repeat library. The output of RepeatModeler was provided to RepeatMasker (Tarailo-Graovac and Chen 2009), along with the RepBase library (Bao, Kojima, and Kohany 2015), to search for various repeat elements in the assembly. PERF (Avvaru, Sowpati, and Mishra 2018) was used to identify simple sequence repeats.

### Gene prediction

For RNA-seq based approach, available paired-end data generated from the transcriptome of female *L*. *boulardi* abdomen (SRR559222) (Goecks *et al.* 2013) was mapped to the assembly using STAR (Dobin *et al.* 2013). The BAM file containing uniquely-mapped read pairs (72% of total reads) was used to construct high-quality transcripts using Cufflinks (Trapnell *et al.* 2013). The same BAM file was submitted for RNA-seq based *ab initio* prediction using BRAKER (Hoff *et al.* 2016). BRAKER uses the RNA-seq data to generate initial gene structures using GeneMark-ET (Lomsadze, Burns, and Borodovsky 2014), and further uses AUGUSTUS (Stanke *et al.* 2008) to predict genes on the generated gene structures. In addition to BRAKER, two other *ab initio* prediction tools were used: GlimmerHMM (Majoros, Pertea, and Salzberg 2004) and SNAP (Korf 2004). Using the gene sets generated from various methods, a final non-redundant set of genes was derived using Evidence Modeler (Haas *et al.* 2008). A protein FASTA file derived using this gene set was further used for functional annotation.

### Gene annotation

BLAST was used to search for homology signatures against SwissProt and TrEMBL databases at an e-value cutoff of 10e-5. InterProScan v5 (Jones *et al.* 2014) was used to search for the homology of protein sequences against various databases such as Pfam, PROSITE, and Gene3D. The gene ontology terms associated with the proteins were retrieved using the InterPro ID assigned to various proteins.

### Mining of homologs

For protein BLAST (blastp), the proteins were used as query sequences to search against *L. boulardi* proteome. The hit with highest e-value was selected as potential ortholog for a given gene and further subjected to Conserved Domains Search using CDD (Marchler-Bauer *et al.* 2017) to look for the presence of specific protein domains. Non-redundant BLAST searches at NCBI database were also done to compare with closely associated species from Hymenoptera and other insect orders.

For translated BLAST (tblastn), the proteins were used as query sequences to search against translated *L. boulardi* genome. The hits with e-value greater than 0.01, irrespective of their percentage identity and alignment length, were used for further analysis. The genomic regions that showed matches in tblastn were extended 5 kb upstream and downstream for gene prediction using GENSCAN. The non-redundant peptides obtained from GENSCAN were then subjected to domain prediction using CDD (Marchler-Bauer *et al.* 2017).

### Multiple sequence alignment and Phylogenetic tree construction

For phylogenetic tree construction of 15 metazoan species, the protein datasets of selected species were downloaded from UniProt (The UniProt Consortium 2019), choosing the non-redundant proteomes wherever possible. Orthologs were obtained and clustered using OrthoFinder (Emms and Kelly 2015). The tree generated by OrthoFinder was visualized using iTOL v3 (Letunic and Bork 2016). For assigning the putative DNA methyltransferases to DNMT1 and DNMT2 subfamily and aligning the chromodomain/chromoshadow domain sequences obtained by tblastn with seed sequences from *D. melanogaster*, Clustal Omega (Madeira *et al.* 2019) was used followed by maximum likelihood tree generation with 1000 bootstrap steps using MEGA (Kumar *et al.* 2018).

### Data availability

The raw reads generated on the Illumina and PacBio platforms are deposited in the Sequence Read Archive (SRA accession SRP144858) of NCBI under the BioProject PRJNA419850. Repeat masked assembly is available on NCBI under the BioProject PRJNA419850 and the accession number is PHTE00000000. Supplemental material available at figshare: https://doi.org/10.25387/g3.11859138.

## Results And Discussion

### Genome assembly and assessment of genome completeness

Previous cytogenetic and karyotypic analysis has estimated the genome size of *L. boulardi* to be around 360Mb (Gokhman *et al.* 2016). We used JellyFish (Marçais and Kingsford 2011) to determine the genome size of *L*. *boulardi* to be 398Mb. Using the five short-read libraries of ∼200X coverage (70.66GB data) and PacBio reads of ∼30X coverage (10.5GB data) (Supplementary file 1: Table S1), MaSuRCA produced an assembly of 375Mb, made of 6341 scaffolds with an N50 of 275Kb (Table 1). MaSuRCA uses both short Illumina reads and long PacBio reads to generate error-corrected super reads, which are further assembled into contigs. It then uses mate-pair information from short-read libraries to scaffold the contigs. The largest scaffold thus obtained was 2.4Mb long, and 50% of the assembly was covered by 380 largest scaffolds (L50). Using GapFiller, 206Kb out of 1.4Mb of N’s could be filled after ten iterations. From this assembly, all scaffolds shorter than 500bp were removed, leaving a total of 6315 scaffolds.

The quality of the genome assembly was measured using two approaches. First, we aligned the paired-end reads generated from a fresh 250bp library to the assembly using bowtie2 (Langmead and Salzberg 2012). 94.64% of the reads could be mapped back, with 92.32% reads mapped in proper pairs. Next, we used BUSCO v3 (Simão *et al.* 2015) to look for the number of single-copy orthologs in the assembly. Out of the 978 BUSCOs in the metazoan dataset, 943 (96.5%) complete BUSCOs were detected in the assembly (Table 2). We also performed BUSCO analysis with the Arthropoda (1066 BUSCOs) and Insecta (1658 BUSCOs) datasets and could identify 97% and 95.7% complete BUSCOs in our assembly, respectively (Table 2). The number of complete Insect BUSCOs present in our assembly was similar to that of the other insect genomes (Supplementary file 1: Table S2). Both the results indicate that the generated assembly was nearly complete, with a good representation of the core gene repertoire with only 2.1% and 3.1% of the Arthropod and Insect specific BUSCOs missing from the assembly respectively.

**Table 2:** BUSCO ANALYSIS FOR ASSESSING THE COMPLETENESS OF GENOME ASSEMBLY

BUSCOs	Lineage		
	Metazoa	Arthropoda	Insecta
Complete Single-Copy	913 (93.4%)	1004 (94.2%)	1538 (92.8%)
Complete Duplicated	30 (3.1%)	30 (2.8%)	48 (2.9%)
Fragmented	11 (1.1%)	10 (0.9%)	20 (1.2%)
Missing	24 (2.4%)	22 (2.1%)	52 (3.1%)
Complete (All)	943 (96.5%)	1034 (97%)	1586 (95.7%)
**Total**	**978**	**1066**	**1658**

### Identification of repeat elements

A total of 868105 repeat elements could be identified using RepeatMasker (Tarailo-Graovac and Chen 2009), covering almost 165Mb (43.88%) of the genome. Table 3 summarizes the number of repeat elements identified in the *L. boulardi* assembly as well as their respective types. We further used PERF (Avvaru, Sowpati, and Mishra 2018) to identify simple sequence repeats of >=12bp length. PERF reported a total of 853,624 SSRs covering 12.24Mb (3.26%) of the genome (Table 4). The density of SSRs in the genome of *L. boulardi* was comparable to other insect genomes (Supplementary file 1: Table S3). Hexamers were the most abundant SSRs (40.1%) in the *L. boulardi* genome, followed by pentamers (15.8%) and monomers (14.3%).

**Table 3:** SUMMARY OF REPEAT ELEMENTS IDENTIFIED BY REPEAT MASKER IN THE GENOME

Repeat Type	Number of Elements	Total Length (bp)	Average Length (bp, rounded)	% Genome Covered
SINEs	3721	1,651,220	444	0.44
LINEs	10573	5,613,129	531	1.49
LTR elements	12312	9,512,954	773	2.53
DNA elements	105817	31,232,845	295	8.31
Unclassified interspersed elements	382214	102,924,940	269	27.39
Small RNA	186	137,204	738	0.04
Satellites	2442	1,028,732	421	0.27
Simple repeats	251669	11,461,332	46	3.05
Low complexity	46977	2,473,942	53	0.66

**Table 4:** DETAILS OF SSRs IDENTIFIED BY PERF IN THE GENOME

Number of SSRs	853,624
Total Repeat bases	12.24Mb
Repeat bases per Mb genome	32,587.49
Number of monomers	122,305 (14.3%)
Number of dimers	101,493 (11.9%)
Number of trimers	72,675 (8.5%)
Number of tetramers	80,493 (9.4%)
Number of pentamers	134,680 (15.8%)
Number of hexamers	341,978 (40.1%)

### Gene prediction and annotation

Coding regions in the assembled genome of *L. boulardi* were predicted using two different approaches: RNA-seq based prediction and *ab initio* prediction. The number of predicted genes using different method is outlined in Table 5. Using the gene sets generated from various methods, a final non-redundant set of 25259 genes was derived using Evidence Modeler (Haas *et al.* 2008) (Table 5). The average gene size in the final gene set is ∼3.9Kb. A protein FASTA file was derived using this gene set, which was further used for functional annotation.

**Table 5:** PREDICTION OF GENES IN *L. Boulardi*: SUMMARY OF VARIOUS METHODS

Evidence Type	Tool	Element	Total Count	Average Length
**RNA-Seq**	Cufflinks	Gene	16930	10216.46
		Exon	86962	404.44
***ab initio***	BRAKER	Gene	45478	2461.26
		Exon	131812	384.35
	GlimmerHMM	Gene	28468	10529.63
		Exon	116583	243.50
	SNAP	Gene	22747	856.46
		Exon	62449	222.72
**Combined**	EvidenceModeler	Gene	25259	3886.27
		Exon	92127	333.69

The functional annotation of predicted proteins was done using a homology-based approach. 11629 and 19795 proteins could be annotated by performing BLAST against SwissProt and TrEMBL databases, respectively. Further, using InterProScan v5 (Jones *et al.* 2014), 12,449 out of 25,259 (49.2%) proteins could be annotated with Pfam, while 9346 and 10952 proteins showed a match in PROSITE and Gene3D databases, respectively (Table 6). The gene ontology terms associated with the proteins were retrieved using the InterPro ID assigned to various proteins. A total of 22729 proteins (89.98%) could be annotated using at least one database.

**Table 6:** GENE ANNOTATION OF THE PREDICTED GENES

Database	Genes Annotated	Percentage Total
SwissProt	11629	46.04
TrEMBL	19795	78.37
Pfam	12449	49.29
Prosite	9346	37.00
Gene3D	10952	43.36
GO	9383	37.15
Annotated	22729	89.98
**Total**	**25259**	**100.00**

### Phylogenetic relationship with Hymenopterans

**Figure 2:**
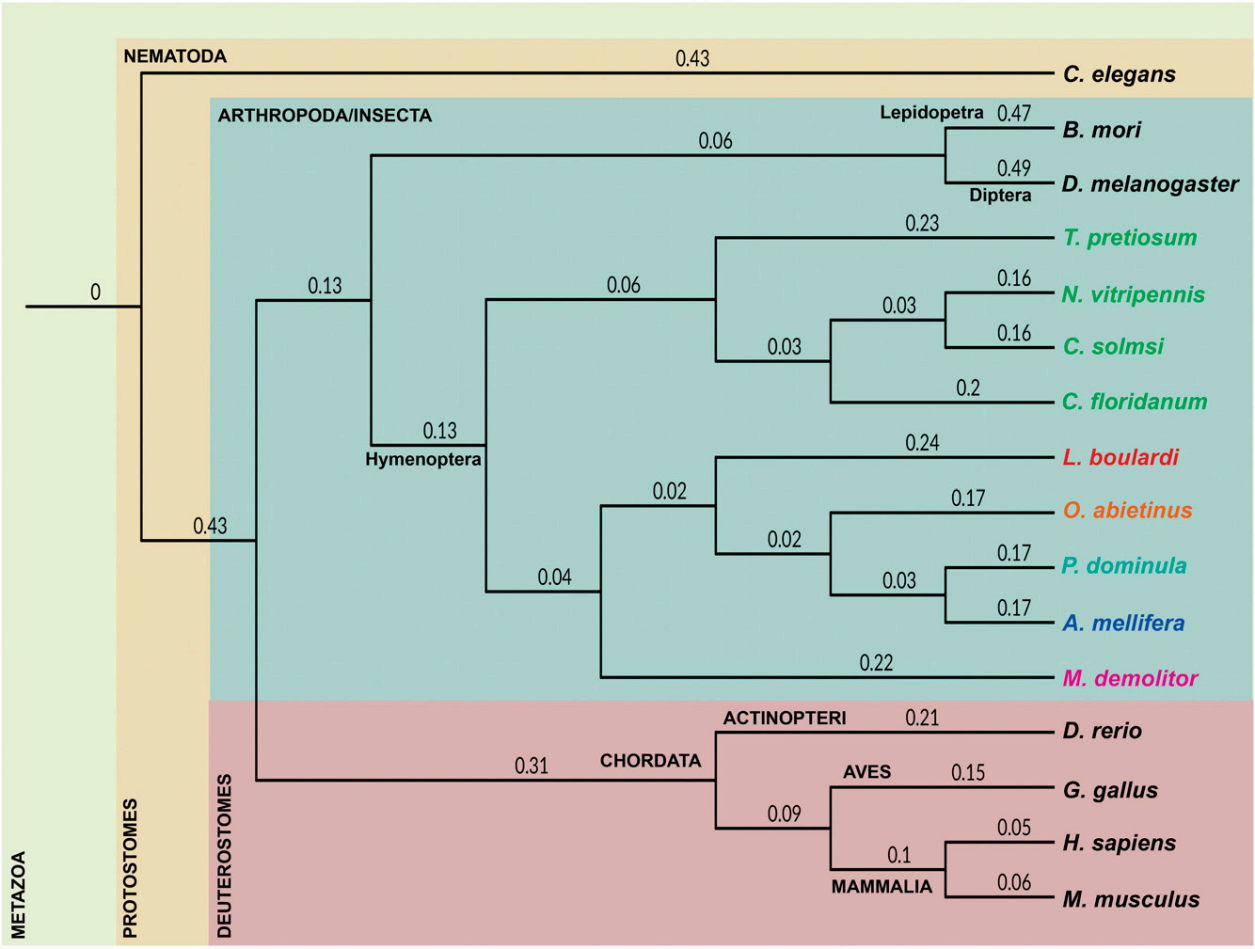
Phylogenetic relationship of *L. boulardi* with selected metazoan species. A phylogenetic tree representing the relationship of *L. boulardi* (red, boldface) with 11 protostomes and four deuterostomes based on 150 single-copy orthologs. Bootstrap values are mentioned at each node. The Phylum/Class is written in uppercase and the order in sentence case. Nine selected species of hymenoptera are shown in different colors based on their superfamily: Orrusoidea (orange), Apoidea (blue), Vespoidea (teal), Chalcidoidea (green), Cynipoidea (red), Ichneumonoidea (magenta).

The evolutionary relationship of *L. boulardi* was examined with fifteen metazoan species: one nematode (*C. elegans*), eleven insects – one dipteran (*D. melanogaster*), one lepidopteran (*B. mori*), seven parasitic hymenopterans (*C. solmsi*, *C. floridanum*, *T. pretiosum*, *N. vitripennis*, *M. demolitor* and *O. abietinus*) and two non-parasitic hymenopterans (*P. dominula*, *A. mellifera*), and four chordates (*D. rerio*, *G. gallus*, *M. musculus* and *H. sapiens*) (Supplementary file 1: Table S4). One hundred fifty single-copy orthologs (Supplementary file 2: Figure S1), were obtained and clustered using OrthoFinder (Emms and Kelly 2015), to understand the phylogenetic relationship between the selected species. The tree generated by OrthoFinder was visualized using iTOL v3 (Letunic and Bork 2016). As expected, *L. boulardi* clusters primarily with Hymenopterans and the phylogeny places it as a separate clade and not with other families of Hymenoptera order (Figure 2).

### Hox genes

Hox genes, a subgroup of Homeobox genes that code for homeodomain-containing transcription factors, play a crucial role during the embryonic development in animals. In addition to their high evolutionary conservation in bilaterian animals, they have received special attention as their genomic arrangement, and expression status determines segment identity along the anterior-posterior body axis (Mallo and Alonso 2013). Unlike vertebrates, where multiple Hox clusters are often found tightly arranged in the genome, the clustering of Hox genes is not very common in invertebrates. The variations observed in the genomic arrangement of Hox genes in insects have helped shed light on the evolution of distinct body plans (Maeda and Karch 2006; Pace, Grbić, and Nagy 2016; Heffer and Pick 2013). Therefore, we investigated the conservation and clustering pattern of Hox genes in the *L. boulardi* genome. The *Drosophila* Hox proteins (Supplementary *file 1: Table S4)* (Miura, Nozawa, and Nei 2011) were used as query sequences in a protein BLAST to search against *L. boulardi* proteome. All the genes except *Ubx* had full-length protein products in EvidenceModeler gene prediction. For *Ubx*, a full-length protein product was detected in the Cufflinks derived dataset obtained from the available transcriptome of *L. boulardi* abdomen (Goecks et al. 2013). In the end, we obtained convincing hits that show high similarity with the Hox proteins of *Drosophila and Hymenopterans (*Supplementary file 1: Table S5 and Supplementary file 3).

The identified Hox genes in *L. boulardi* are spread across four scaffolds. The bithorax complex orthologs – *Ubx*, *abd-A* and *Abd-B* – are located on scaffold00039 (780Kb). However, the orthologs of Antennapedia complex (ANT-C) are distributed in three scaffolds – *pb* and *lab* are located in scaffold00168 (454Kb), *Scr* and *Dfd* are located in scaffold00375 (278Kb), and scaffold00572 (196Kb) contains *Antp*. Overall, the Hox genes are well conserved in *L. boulardi*, span around 1.7Mb of the genome (assuming the scaffolds are contiguous) and are not tightly clustered. To further examine the degree to which Hox genes are dispersed in the genome, the scaffold level draft genome has to be assembled at a chromosome level using techniques such as chromosome linkage mapping, optical mapping, or targeted sequencing of BACs.

### Sex determination genes

Hymenopterans have a haplodiploid sex-determination system wherein the females are diploid, and males are haploid. The diploid females develop from fertilized eggs, whereas the unfertilized eggs give rise to haploid males (arrhenotoky) (Heimpel and de Boer 2008). The two major experimentally supported paradigms of sex determination in Hymenopterans are complementary sex determination (CSD) (Beye *et al.* 2003) and genome imprinting (Dobson and Tanouye 1998). It has been reported in previous studies that *Leptopilina* genus lacks CSD (Hey and Gargiulo 1985; Biémont and Bouletreau 1980; Van Wilgenburg, Driessen, and Beukeboom 2006) but whether the primary signal for sex determination cascade is the differential methylation status of the maternal and paternal chromosome, is still unclear.

We took the previously described sex determination proteins downstream in the cascade from *D. melanogaster* and *L. clavipes* (Geuverink *et al.* 2018) and searched for their homologs in *L. boulardi* using blastp approach. We found putative orthologs of the major effector genes (*doublesex* and *fruitless*) and the genes regulating their sex-specific splicing (*transformer* and *transformer-2*) (Supplementary file 1: Table S6), implying that the downstream cascade of sex determination is well preserved. However, we could only identify one transformer gene as opposed to the presence of transformer and its paralogue *transformerB* in L. clavipes.

### DNA methyltransferases

Two families of DNA methyltransferases (DNMTs) are well-known to be responsible for DNA methylation, which occurs primarily at CpG sites in mammals. DNMT3 is a *de novo* methyltransferase, while DNMT1 is known to be involved in the maintenance of DNA methylation (Goll and Bestor 2005). DNMT2, on the other hand, the most conserved methyltransferase in eukaryotes, was initially assigned as a member of DNMT family but later renamed as TRDMT1 (tRNA aspartic acid methyltransferase 1) that justifies its negligible contribution to the DNA methylome (Jurkowski *et al.* 2008). Other than the role in caste development in social insects (Chittka, Wurm, and Chittka 2012; Herb *et al.* 2012; Bonasio *et al.* 2012), cytosine methylation (genome imprinting) has been shown to be the primary signal of the sex determination cascade in the haplodiploid hymenopterans lacking complementary sex determination system (Dobson and Tanouye 1998). In order to assess the methylation status of *L. boulardi* genome, we looked at the CpG content in all exons. Typically, exons that undergo methylation in the genome display an underrepresentation of CpG content due to spontaneous deamination of methylated cytosines into thymines. Hence, genomes that have DNA methylation show a bimodal distribution of CpG content (Elango and Yi 2008), as shown for human and honey bee exons in Supplementary file 2: Figure S2A and B. We observed no such bimodality for exons of *L. boulardi* (Supplementary file 2: Figure S2C).

**Figure 3:**
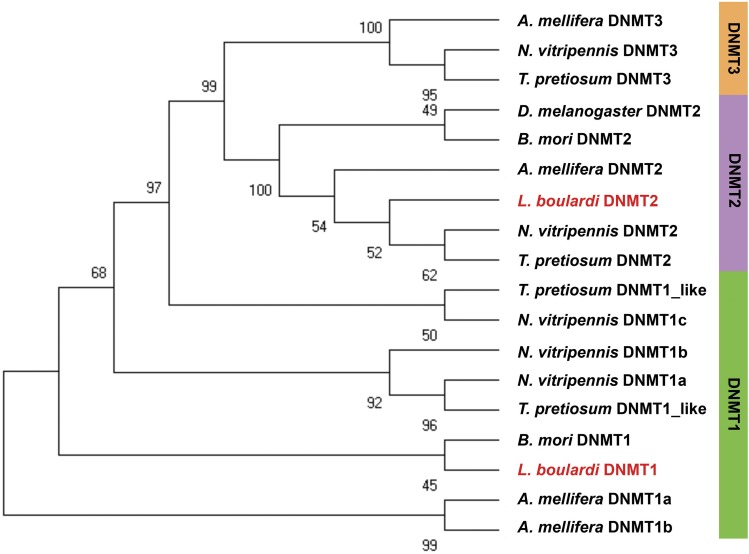
Phylogram of putative DNA methyltransferases in *Leptopilina boulardi*. *L. boulardi* is written in red font (boldface). Bootstrap values are shown at each node. Putative DNMT2 and DNMT1 of *L. boulardi* clusters with the DNMT2 and DNMT1 of other insects, respectively.

We further searched for the presence of DNA methyltransferases in *L. boulardi*. Corresponding sequences from *N. vitripennis* (Supplementary file 1: Table S7) were used as seed sequences for identification of DNMTs in *L. boulardi* using blastp and tblastn. We obtained two putative DNA methyltransferases, which were then aligned to DNMTs from *A. mellifera*, *Bombyx mori*, *D. melanogaster*, *N. vitripennis* and *T. pretiosum* (Supplementary file 1: Table S6) using Clustal Omega (Madeira *et al.* 2019). The maximum likelihood tree thus generated with 1000 bootstrap steps using MEGA (Kumar *et al.* 2018) assigned the two putative DNA methyltransferases to DNMT1 and DNMT2 subfamily (Figure 3, and Supplementary file 4).

An incomplete set of DNA methylation toolkit has been previously described in insects, and it does not always mean an absence of DNA methylation (Glastad *et al.* 2014; Bewick *et al.* 2017). The unimodal distribution of observed/expected CpG content in the exons and the presence DNMT1 hints toward possible DNA methylation in non-CpG context in the genome of *L. boulardi*. However, the presence of detectable levels of DNA methylation and the methylation pattern in this genome of *L. boulardi* at different developmental stages needs to be further investigated experimentally.

### Polycomb group, Trithorax group and heterochromatin factors

The expression of genes in the eukaryotes is regulated by numerous evolutionary conserved factors that act in a complex to either direct post-translational modifications of histones or remodel chromatin in an ATP-dependent manner (Grimaud, Nègre, and Cavalli 2006). It is well established that the transcriptionally active states of chromatin are maintained by Trithorax group (TrxG) of proteins. In contrast, Polycomb group (PcG) of proteins and heterochromatin factors maintain the transcriptionally repressed state of chromatin (facultative and constitutive heterochromatin, respectively). Together, they are critical for the establishment and maintenance of chromatin states throughout the development of eukaryotes (Ringrose and Paro 2004; Allshire and Madhani 2018; Schotta 2002). We examined the conservation of these factors in the genome of *L*. *boulardi*. Protein sequences from *D. melanogaster* were used as query sequences in a standalone BLAST to search against *L. boulardi* protein data set. The Polycomb group (PcG) and Trithorax group (TrxG) of proteins are well conserved in *L. boulardi* (Supplementary file 1: Table S8). However, unlike *Drosophila*, *polyhomeotic*, *extra sexcombs*, *pleiohomeotic* is present in only one copy. Heterochromatin factors, Heterochromatin protein 1 (HP1) family and Suppressor of variegation 3-9 (Su(var)3-9), the proteins that bind to and introduce heterochromatic histone methylation, respectively, are also conserved. Still, only one full length HP1 could be identified using blastp.

We further did a tblastn for identification of chromodomain and chromoshadow domain containing proteins in *L. boulardi*, since the characteristic feature of HP1 protein family is the presence of an N-terminal chromodomain and a C-terminal chromoshadow domain (Renato Paro 1990; R. Paro and Hogness 1991; Assland and Stewart 1995). All known chromodomain and chromoshadow domain sequences from *D. melanogaster* were used as seed sequences. A total of 49 proteins containing chromodomain were identified, which falls into four classes (Supplementary file 1: Table S9). All the chromodomain/chromoshadow domain sequences obtained were aligned with seed sequences from *D. melanogaster* using Clustal Omega (Madeira *et al.* 2019) followed by maximum likelihood tree generation with 1000 bootstrap steps using MEGA (Kumar *et al.* 2018) (Supplementary file 2: Figure S3 and S4). We identified one HP1 protein (Class I) containing a chromodomain followed by a chromoshadow domain and eight proteins containing a single chromodomain (Class II). Four out of 49 have paired tandem chromodomain (Class IV) and 36 proteins contain chromodomain in combination with other domain families (Class III). A similar analysis done previously in ten hymenopterans has reported that Hymenopterans have a simple HP1 gene family comprising of one full HP1 and two partial HP1 genes (Fang, Schmitz, and Ferree 2015). However, we identified one full (chromodomain and chromoshadow domain) and eight partial (only chromodomain) HP1 homologs. The full HP1 protein identified is more similar to *HP1b* of *D. melanogaster* than to other paralogs. This indicates that the HP1 is more dynamic in *L. boulardi* than what is reported earlier in other hymenopterans.

## Conclusions

*Leptopilina* has been extensively used as a model system to study host-parasitoid biology. Our study presents a high-quality reference genome (375 Mb) of the specialist parasitoid wasp *Leptopilina boulardi* showing almost a complete coverage of the core gene repertoire shown by BUSCO analysis. A total of 25,259 protein-coding genes were predicted, out of which 22729 could be annotated using known protein signatures. We show that the genes responsible for determining the anteroposterior body axis (*Hox* genes) and sex determination are well conserved. *L. boulardi* has an incomplete DNA methylation toolkit; it is devoid of a *de novo* DNA methyltransferase (DNMT3). The HP1 family is much more diverse as compared to other hymenopterans. The other epigenetic regulators, Polycomb and trithorax group of proteins, are also retained. Overall, the basic machinery of epigenetic regulation is conserved, and though the unique features are noticed, their relevance needs further investigations.

The *L. boulardi* genome reported in this study provides a valuable resource to researchers studying parasitoids and can help shed light on the mechanisms of host-parasitoid interactions and understanding the immune response mechanisms in insects. The genome sequence of *L. boulardi* will also be a key element in understanding the evolution of parasitism in figitids. It will further enable genome editing and thereby advance the genetics of *L. boulardi*, facilitate the comparative studies of *Drosophila* parasitoids. More importantly, this resource fulfils the prerequisite for initiating research on epigenetic mechanisms underlying parasitism, and sex determination and other developmental mechanisms in *Leptopilina* genus.

## References

[bib1] Allshire R. C., and Madhani H. D., 2018 Ten Principles of Heterochromatin Formation and Function. Nat. Rev. Mol. Cell Biol. 19: 229–244. 10.1038/nrm.2017.119 29235574 PMC6822695

[bib2] Assland R., and Stewart F., 1995 The Chromo Shadow Domain, a Second Chromo Domain in Heterochromatin-Binding Protein 1, HP1. Nucleic Acids Res. 23: 3168–3173. 10.1093/nar/23.16.3168 7667093 PMC307174

[bib3] Avvaru A. K., Sowpati D. T., and Mishra R. K., 2018 PERF: An Exhaustive Algorithm for Ultra-Fast and Efficient Identification of Microsatellites from Large DNA Sequences. Bioinformatics 34: 943–948. 10.1093/bioinformatics/btx721 29121165

[bib4] Bao W., Kojima K. K., and Kohany O., 2015 Repbase Update, a Database of Repetitive Elements in Eukaryotic Genomes. Mob. DNA 6: 11 10.1186/s13100-015-0041-9 26045719 PMC4455052

[bib5] The UniProt Consortium 2019 UniProt: A Worldwide Hub of Protein Knowledge. Nucleic Acids Res. 47: D506–D515. 10.1093/nar/gky1049 30395287 PMC6323992

[bib6] Benson G., 1999 Tandem Repeats Finder: A Program to Analyze DNA Sequences. Nucleic Acids Res. 27: 573–580. http://www.ncbi.nlm.nih.gov/pubmed/9862982. 10.1093/nar/27.2.573 9862982 PMC148217

[bib7] Bewick A. J., Vogel K. J., Moore A. J., and Schmitz R. J., 2017 Evolution of DNA Methylation across Insects. Mol. Biol. Evol. 34: 654–655. 10.1093/molbev/msw264 28025279 PMC5400375

[bib8] Beye M., Hasselmann M., Fondrk M. K., Page R. E., and Omhol S. W., 2003 The Gene Csd Is the Primary Signal for Sexual Development in the Honeybee and Encodes an SR-Type Protein. Cell 114: 419–429. 10.1016/S0092-8674(03)00606-8 12941271

[bib9] Biémont C., and Bouletreau M., 1980 Hybridization and Inbreeding Effects on Genome Coadaptation in a Haplo-Diploid Hymenoptera:Cothonaspis Boulardi (Eucoilidae). Experientia 36: 45–47. 10.1007/BF02003961

[bib10] Bonasio R., Li Q., Lian J., Mutti N. S., Jin L., 2012 Genome-Wide and Caste-Specific DNA Methylomes of the Ants Camponotus Floridanus and Harpegnathos Saltator. Curr. Biol. 22: 1755–1764. 10.1016/j.cub.2012.07.042 22885060 PMC3498763

[bib11] Boulétreau M., and Wajnberg E., 1986 Comparative Responses of Two Sympatric Parasitoid Cynipids to the Genetic and Epigenetic Variations of the Larvae of Their Host, Drosophila Melanogaster. Entomol. Exp. Appl. 41: 107–114. 10.1111/j.1570-7458.1986.tb00516.x

[bib12] Chittka A., Wurm Y., and Chittka L., 2012 Epigenetics: The Making of Ant Castes. Curr. Biol. 22: R835–R838. 10.1016/j.cub.2012.07.045 23058801

[bib13] Colinet D., Schmitz A., Cazes D., Jean-luc G., and Poirié M., 2010 The Origin of Intraspecific Variation of Virulence in an Eukaryotic Immune Suppressive Parasite. PLoS Pathog. 6: e1001206 10.1371/journal.ppat.1001206 21124871 PMC2991256

[bib14] Dobin A., Davis C. A., Schlesinger F., Drenkow J., Zaleski C., 2013 STAR: Ultrafast Universal RNA-Seq Aligner. Bioinformatics 29: 15–21. 10.1093/bioinformatics/bts635 23104886 PMC3530905

[bib15] Dobson S. L., and Tanouye M. A. 1998 Evidence for a Genomic Imprinting Sex Determination Mechanism in Nasonia Vitripennis (Hymenoptera; Chalcidoidea). Genetics 149: 233–242. http://www.ncbi.nlm.nih.gov/pubmed/9584099. 9584099 10.1093/genetics/149.1.233 PMC1460136

[bib16] Dubuffet A., Doury G., Labrousse C., Drezen J.-M., Carton Y., 2008 Variation of Success of Leptopilina Boulardi in Drosophila Yakuba: The Mechanisms Explored. Dev. Comp. Immunol. 32: 597–602. 10.1016/j.dci.2007.10.009 18061668

[bib17] Dupas S, Brehelin M, Frey F, and Carton Y 1996 Immune Suppressive Virus-like Particles in a Drosophila Parasitoid: Significance of Their Intraspecific Morphological Variations. Parasitology 113: 207–212. 10.1017/S0031182000081981 8811846

[bib18] Eggleton P., and Belshaw R., 1992 Insect Parasitoids: An Evolutionary Overview. Philos. Trans. R. Soc. Lond. B Biol. Sci. 337: 1–20. 10.1098/rstb.1992.0079

[bib19] Elango N., and Yi S. V., 2008 DNA Methylation and Structural and Functional Bimodality of Vertebrate Promoters. Mol. Biol. Evol. 25: 1602–1608. 10.1093/molbev/msn110 18469331

[bib20] Emms D. M., and Kelly S., 2015 OrthoFinder: Solving Fundamental Biases in Whole Genome Comparisons Dramatically Improves Orthogroup Inference Accuracy. Genome Biol. 16: 157 10.1186/s13059-015-0721-2 26243257 PMC4531804

[bib21] Fang C., Schmitz L., and Ferree P M 2015 An Unusually Simple HP1 Gene Set in Hymenopteran Insects. Biochem. Cell Biol. 93: 596–603. 10.1139/bcb-2015-0046 26419616

[bib22] Fellowes M. D. E., and Godfray H. C. J., 2000 The Evolutionary Ecology of Resistance to Parasitoids by Drosophila. Heredity 84: 1–8. 10.1046/j.1365-2540.2000.00685.x 10692005

[bib23] Fleury F., Gibert P., Ris N., and Allemand R., 2009 Ecology and Life History Evolution of Frugivorous Drosophila Parasitoids. Adv. Parasitol. 70: 3–44. 10.1016/S0065-308X(09)70001-6 19773065

[bib24] Geuverink E., Kraaijeveld K., van Leussen M., Chen F., Pijpe J., 2018 Evidence for Involvement of a Transformer Paralogue in Sex Determination of the Wasp Leptopilina Clavipes. Insect Mol. Biol. 27: 780–795. 10.1111/imb.12522 30039559

[bib25] Glastad K. M., Hunt B. G., and Goodisman M. A. D., 2014 Evolutionary Insights into DNA Methylation in Insects. Curr. Opin. Insect Sci. 1: 25–30. 10.1016/j.cois.2014.04.001 32846726

[bib26] Godfray H. C. J., 1994 Parasitoids : Behavioral and Evolutionary Ecology, Princeton University Press, NY 10.1515/9780691207025

[bib27] Goecks J., Mortimer N. T., Mobley J. A., Bowersock G. J., Taylor J., 2013 Integrative Approach Reveals Composition of Endoparasitoid Wasp Venoms. PLoS One 8: e64125 10.1371/journal.pone.0064125 23717546 PMC3662768

[bib28] Gokhman V. E., Bolsheva N. L., Govind S., and Muravenko O. V., 2016 A Comparative Cytogenetic Study of Drosophila Parasitoids (Hymenoptera, Figitidae) Using DNA-Binding Fluorochromes and FISH with 45S RDNA Probe. Genetica 144: 335–339. 10.1007/s10709-016-9902-5 27150102

[bib29] Goll M. G., and Bestor T. H., 2005 EUKARYOTIC CYTOSINE METHYLTRANSFERASES. Annu. Rev. Biochem. 74: 481–514. 10.1146/annurev.biochem.74.010904.153721 15952895

[bib30] Grimaud C., Nègre N., and Cavalli G., 2006 From Genetics to Epigenetics: The Tale of Polycomb Group and Trithorax Group Genes. Chromosome Res. 14: 363–375. 10.1007/s10577-006-1069-y 16821133

[bib31] Gueguen G., Rajwani R., Paddibhatla I., Morales J., and Govind S., 2011 VLPs of Leptopilina Boulardi Share Biogenesis and Overall Stellate Morphology with VLPs of the Heterotoma Clade. Virus Res. 160: 159–165. 10.1016/j.virusres.2011.06.005 21704090 PMC3905611

[bib32] Haas B. J., Salzberg S. L., Zhu W., Pertea M., Allen J. E., 2008 Automated Eukaryotic Gene Structure Annotation Using EVidenceModeler and the Program to Assemble Spliced Alignments. Genome Biol. 9: R7 10.1186/gb-2008-9-1-r7 18190707 PMC2395244

[bib33] Heath B. D., Butcher R. D., Whitfield W. G., and Hubbard S. F., 1999 Horizontal Transfer of Wolbachia between Phylogenetically Distant Insect Species by a Naturally Occurring Mechanism. Curr. Biol. 9: 313–316. 10.1016/S0960-9822(99)80139-0 10209097

[bib34] Heffer A., and Pick L., 2013 Conservation and Variation in Hox Genes: How Insect Models Pioneered the Evo-Devo Field. Annu. Rev. Entomol. 58: 161–179. 10.1146/annurev-ento-120811-153601 23317041

[bib35] Heimpel G. E., and de Boer J. G., 2008 Sex Determination in the Hymenoptera. Annu. Rev. Entomol. 53: 209–230. 10.1146/annurev.ento.53.103106.093441 17803453

[bib36] Heraty, J., 2009 Parasitoid Biodiversity and Insect Pest Management. In *Insect Biodiversity*, edited by Robert G. Foottit and Peter H. Adler, 2nd ed., I:445–62. Oxford, UK: Wiley-Blackwell. 10.1002/9781444308211.ch19

[bib37] Herb B. R., Wolschin F., Hansen K. D., Aryee M. J., Ben Langmead R. I., 2012 Reversible Switching between Epigenetic States in Honeybee Behavioral Subcastes. Nat. Neurosci. 15: 1371–1373. 10.1038/nn.3218 22983211 PMC3518384

[bib38] Hey J., and Gargiulo M. K., 1985 Sex-Ratio Changes in Leptopilina Heterotoma in Response to Inbreeding. J. Hered. 76: 209–211. 10.1093/oxfordjournals.jhered.a110069

[bib39] Hita M. T., Poirié M., Leblanc N., Lemeunier F., Lutcher F., 1999 Genetic Localization of a Drosophila Melanogaster Resistance Gene to a Parasitoid Wasp and Physical Mapping of the Region. Genome Res. 9: 471–481. 10.1101/gr.9.5.471 10330127 PMC310767

[bib40] Hoff K. J., Lange S., Lomsadze A., Borodovsky M., and Stanke M., 2016 BRAKER1: Unsupervised RNA-Seq-Based Genome Annotation with GeneMark-ET and AUGUSTUS: Table 1. Bioinformatics 32: 767–769. 10.1093/bioinformatics/btv661 26559507 PMC6078167

[bib41] Howell L., Sampson C. J., Xavier M. J., Bolukbasi E., Heck M. M., and Williams M. J. 2012 A Directed Miniscreen for Genes Involved in the Drosophila Anti-Parasitoid Immune Response. Immunogenetics 64: 155–161. 10.1007/s00251-011-0571-3 21947570

[bib42] Jones P., Binns D., Chang H. Y., Fraser M., Li W., 2014 InterProScan 5: Genome-Scale Protein Function Classification. Bioinformatics 30: 1236–1240. 10.1093/bioinformatics/btu031 24451626 PMC3998142

[bib43] Jurkowski T. P., Meusburger M., Phalke S., Helm M., Nellen W., 2008 Human DNMT2 Methylates TRNAAsp Molecules Using a DNA Methyltransferase-like Catalytic Mechanism. RNA 14: 1663–1670. 10.1261/rna.970408 18567810 PMC2491481

[bib44] Kaiser L., Couty A., and Perez-Maluf R., 2009 Chapter 3 Dynamic Use of Fruit Odours to Locate Host Larvae: Individual Learning, Physiological State and Genetic Variability as Adaptive Mechanisms. Advances in Parasitology 70: 67–95. 10.1016/S0065-308X(09)70003-X 19773067

[bib45] Korf I., 2004 Gene Finding in Novel Genomes. BMC Bioinformatics 5: 59 10.1186/1471-2105-5-59 15144565 PMC421630

[bib46] Kraaijeveld A R, Van Alphen J. J., and Godfray H. C. 1998 The Coevolution of Host Resistance and Parasitoid Virulence. Parasitology 116: S29–S45. 10.1017/S0031182000084924 9695108

[bib47] Kraaijeveld, Alex R., and H. Charles J. Godfray. 2009 “Chapter 10 Evolution of Host Resistance and Parasitoid Counter-Resistance.” In *Advances in Parasitology*, 1st ed., 70: 257–80. Elsevier Ltd. 10.1016/S0065-308X(09)70010-7 19773074

[bib48] Kraaijeveld K., Anvar S. Y., Frank J., Schmitz A., Bast J., 2016 Decay of Sexual Trait Genes in an Asexual Parasitoid Wasp. Genome Biol. Evol. 8: 3685–3695. 10.1093/gbe/evw273 28172869 PMC5381511

[bib49] Kruitwagen A., Beukeboom L. W., and Wertheim B., 2018 Optimization of Native Biocontrol Agents, with Parasitoids of the Invasive Pest Drosophila Suzukii as an Example. Evol. Appl. 11: 1473–1497. 10.1111/eva.12648 30344621 PMC6183459

[bib50] Kumar S., Stecher G., Li M., Knyaz C., and Tamura K., 2018 MEGA X: Molecular Evolutionary Genetics Analysis across Computing Platforms. Mol Biol Evol 35: 1547–1549. 10.1093/molbev/msy096 29722887 PMC5967553

[bib51] Langmead B., and Salzberg S. L. 2012 Fast Gapped-Read Alignment with Bowtie 2. Nat. Methods 9: 357–359. 10.1038/nmeth.1923 22388286 PMC3322381

[bib52] LaSalle J., and Gauld I., 1991 Parasitic Hymenoptera and the Biodiversity Crisis. Redia (Firenze) 74: 515–334. http://scholar.google.com/scholar?hl=en&btnG=Search&q=intitle:Parasitic+Hymenoptera+and+the+biodiversity+crisis#0.

[bib53] Lee M. J., Kalamarz M. E., Paddibhatla I., Small C., Rajwani R., 2009 Virulence Factors and Strategies of Leptopilina Spp.: Selective Responses in Drosophila Hosts. Advances in Parasitology, Vol. 70, Ed. 1st chap 5. Elsevier Ltd., Netherlands 10.1016/S0065-308X(09)70005-3 PMC3363966 19773069

[bib54] Lepetit D., Gillet B., Hughes S., Kraaijeveld K., and Varaldi J., 2017 Genome Sequencing of the Behaviour Manipulating Virus LbFV Reveals a Possible New Virus Family. Genome Biol. Evol. 8: evw277 10.1093/gbe/evw277 PMC5381508 28173110

[bib55] Letunic I., and Bork P., 2016 Interactive Tree of Life (ITOL) v3: An Online Tool for the Display and Annotation of Phylogenetic and Other Trees. Nucleic Acids Res. 44: W242–W245. 10.1093/nar/gkw290 27095192 PMC4987883

[bib56] Lomsadze A., Burns P. D., and Borodovsky M., 2014 Integration of Mapped RNA-Seq Reads into Automatic Training of Eukaryotic Gene Finding Algorithm. Nucleic Acids Res. 42: e119 10.1093/nar/gku557 24990371 PMC4150757

[bib57] Figueiredo L. C., Cruz I., Silva R. B., and Foster J. E., 2015 Biological Control with Trichogramma Pretiosum Increases Organic Maize Productivity by 19.4%. Agron. Sustain. Dev. 35: 1175–1183. 10.1007/s13593-015-0312-3

[bib58] Machtinger E. T., Geden C. J., Kaufman P. E., and House A. M., 2015 Use of Pupal Parasitoids as Biological Control Agents of Filth Flies on Equine Facilities. J. Integr. Pest Manag. 6: 16 10.1093/jipm/pmv015

[bib59] Madeira F., Park Y. M., Lee J., Buso N., Gur T., 2019 The EMBL-EBI Search and Sequence Analysis Tools APIs in 2019. Nucleic Acids Res. 47: W636–W641. 10.1093/nar/gkz268 30976793 PMC6602479

[bib60] Maeda R. K., and Karch F., 2006 The ABC of the BX-C: The Bithorax Complex Explained. Development 133: 1413–1422. 10.1242/dev.02323 16556913

[bib61] Majoros W. H., Pertea M., and Salzberg S. L., 2004 TigrScan and GlimmerHMM: Two Open Source Ab Initio Eukaryotic Gene-Finders. Bioinformatics 20: 2878–2879. 10.1093/bioinformatics/bth315 15145805

[bib62] Mallo M., and Alonso C. R., 2013 The Regulation of Hox Gene Expression during Animal Development. Development 140: 3951–3963. 10.1242/dev.068346 24046316

[bib63] Marçais G., and Kingsford C., 2011 A Fast, Lock-Free Approach for Efficient Parallel Counting of Occurrences of k-Mers. Bioinformatics 27: 764–770. 10.1093/bioinformatics/btr011 21217122 PMC3051319

[bib64] Marchler-Bauer A., Bo Y., Han L., He J., Lanczycki C. J., 2017 CDD/SPARCLE: Functional Classification of Proteins via Subfamily Domain Architectures. Nucleic Acids Res. 45: D200–D203. 10.1093/nar/gkw1129 27899674 PMC5210587

[bib65] Martínez G., González A., and Dicke M., 2018 Rearing and Releasing the Egg Parasitoid Cleruchoides Noackae, a Biological Control Agent for the Eucalyptus Bronze Bug. Biol. Control 123: 97–104. 10.1016/j.biocontrol.2018.05.008

[bib66] Martinez J., Duplouy A., Woolfit M., Vavre F., O’Neill S. L., 2012 Influence of the Virus LbFV and of Wolbachia in a Host-Parasitoid Interaction. PLoS One 7: e35081 10.1371/journal.pone.0035081 22558118 PMC3338833

[bib67] Mayjonade B., Gouzy J., Donnadieu C., Pouilly N., Marande W., 2016 Extraction of High-Molecular-Weight Genomic DNA for Long-Read Sequencing of Single Molecules. Biotechniques 61: 203–205. 10.2144/000114460 27712583

[bib68] Miura S., Nozawa M., and Nei M., 2011 Evolutionary Changes of the Target Sites of Two MicroRNAs Encoded in the Hox Gene Cluster of Drosophila and Other Insect Species. Genome Biol. Evol. 3: 129–139. 10.1093/gbe/evq088 21187351 PMC3041157

[bib69] Nadalin Francesca, Vezzi Francesco, and Policriti Alberto 2012 GapFiller: A de Novo Assembly Approach to Fill the Gap within Paired Reads. BMC Bioinformatics 13: S8 10.1186/1471-2105-13-S14-S8 PMC3439727 23095524

[bib70] Pace R. M., Grbić M., and Nagy L. M., 2016 Composition and Genomic Organization of Arthropod Hox Clusters. Evodevo 7: 11 10.1186/s13227-016-0048-4 27168931 PMC4862073

[bib71] Paro R., and Hogness D. S., 1991 The Polycomb Protein Shares a Homologous Domain with a Heterochromatin-Associated Protein of Drosophila. Proc. Natl. Acad. Sci. USA 88: 263–267. 10.1073/pnas.88.1.263 1898775 PMC50790

[bib72] Paro R., 1990 Imprinting a Determined State into the Chromatin of Drosophila. Trends Genet. 6: 416–421. 10.1016/0168-9525(90)90303-N 1982376

[bib73] Patot S., Allemand R., Fleury F., and Varaldi J., 2012 An Inherited Virus Influences the Coexistence of Parasitoid Species through Behaviour Manipulation. Ecol. Lett. 15: 603–610. 10.1111/j.1461-0248.2012.01774.x 22487404

[bib74] Patot S., Lepetit D., Charif D., Varaldi J., and Fleury F., 2009 Molecular Detection, Penetrance, and Transmission of an Inherited Virus Responsible for Behavioral Manipulation of an Insect Parasitoid. Appl. Environ. Microbiol. 75: 703–710. 10.1128/AEM.01778-08 19060167 PMC2632122

[bib75] Price Alkes L., Jones Neil C., and Pevzner Pavel A. 2005 De Novo Identification of Repeat Families in Large Genomes. Bioinformatics 21: i351–i358. 10.1093/bioinformatics/bti1018 15961478

[bib76] Ringrose L., and Paro R., 2004 Epigenetic Regulation of Cellular Memory by the Polycomb and Trithorax Group Proteins. Annu. Rev. Genet. 38: 413–443. 10.1146/annurev.genet.38.072902.091907 15568982

[bib77] Rizki R. M., and Rizki T. M., 1990 Parasitoid Virus-like Particles Destroy Drosophila Cellular Immunity. Proc. Natl. Acad. Sci. USA 87: 8388–8392. 10.1073/pnas.87.21.8388 2122461 PMC54961

[bib78] Salazar-Jaramillo L., Jalvingh K. M., de Haan A., Kraaijeveld K., Buermans H., 2017 Inter- and Intra-Species Variation in Genome-Wide Gene Expression of Drosophila in Response to Parasitoid Wasp Attack. BMC Genomics 18: 331 10.1186/s12864-017-3697-3 28449654 PMC5406980

[bib79] Schlenke T. A., Morales J., Govind S., and Clark A. K., 2007 Contrasting Infection Strategies in Generalist and Specialist Wasp Parasitoids of Drosophila Melanogaster. PLoS Pathog. 3: 1486–1501. 10.1371/journal.ppat.0030158 17967061 PMC2042021

[bib80] Schotta G., 2002 Central Role of Drosophila SU(VAR)3–9 in Histone H3–K9 Methylation and Heterochromatic Gene Silencing. EMBO J. 21: 1121–1131. 10.1093/emboj/21.5.1121 11867540 PMC125909

[bib81] Simão F. A., Waterhouse R. M., Ioannidis P., Kriventseva E. V., and Zdobnov E. M., 2015 BUSCO: Assessing Genome Assembly and Annotation Completeness with Single-Copy Orthologs. Bioinformatics 31: 3210–3212. 10.1093/bioinformatics/btv351 26059717

[bib82] Small C., Paddibhatla I., Rajwani R., and Govind S., 2012 An Introduction to Parasitic Wasps of Drosophila and the Antiparasite Immune Response. J. Vis. Exp. e3347 10.3791/3347 PMC3466936 22588641

[bib83] Smith S. M., 1996 Biological Control with Trichogramma: Advances, Successes, and Potential of Their Use. Annu. Rev. Entomol. 41: 375–406. 10.1146/annurev.en.41.010196.002111 15012334

[bib84] Stanke M., Diekhans M., Baertsch R., and Haussler D., 2008 Using Native and Syntenically Mapped CDNA Alignments to Improve de Novo Gene Finding. Bioinformatics 24: 637–644. 10.1093/bioinformatics/btn013 18218656

[bib85] Tarailo-Graovac, M., and N. Chen, 2009 “Using RepeatMasker to Identify Repetitive Elements in Genomic Sequences.” In *Current Protocols in Bioinformatics*, **Chapter 4**: Unit 4.10: John Wiley & Sons, Hoboken, NJ. 10.1002/0471250953.bi0410s25 19274634

[bib86] Tracy Reynolds K., and Hardy I. C. W., 2004 Superparasitism: A Non-Adaptive Strategy? Trends Ecol. Evol. 19: 347–348. 10.1016/j.tree.2004.03.021 16701282

[bib87] Trapnell C., Hendrickson D. G., Sauvageau M., Goff L., Rinn J. L., 2013 Differential Analysis of Gene Regulation at Transcript Resolution with RNA-Seq. Nat. Biotechnol. 31: 46–53. 10.1038/nbt.2450 23222703 PMC3869392

[bib88] Varaldi J., and Lepetit D., 2018 Deciphering the Behaviour Manipulation Imposed by a Virus on Its Parasitoid Host: Insights from a Dual Transcriptomic Approach. Parasitology 145: 1979–1989. 10.1017/S0031182018000835 29806574

[bib89] Varaldi J., Fouillet P., Ravallec M., López-Ferber M., Boulétreau M., 2003 Infectious Behavior in a Parasitoid. Science 302: 1930 10.1126/science.1088798 14564013

[bib90] Varaldi J., Patot S., Nardin M., and Gandon S., 2009 Chapter 13 A Virus-Shaping Reproductive Strategy in a Drosophila Parasitoid. Advances in Parasitology 70: 333–363. 10.1016/S0065-308X(09)70013-2 19773077

[bib91] Vavre F., Mouton L., and Pannebakker B. A., 2009 Chapter 12 Drosophila–Parasitoid Communities as Model Systems for Host–Wolbachia Interactions. Advances in Parasitology 70: 299–331. 10.1016/S0065-308X(09)70012-0 19773076

[bib92] Werren, J. H., D. Windsor, and L. Guo, 1995 “Distribution of Wolbachia among Neotropical Arthropods.” Edited by Kristin Michel. *Proceedings of the Royal Society of London. Series B: Biological Sciences* 262 (1364): 197–204. 10.1098/rspb.1995.0196. 10.1098/rspb.1995.0196

[bib93] van Wilgenburg E. V., Driessen G., and Beukeboom L. W., 2006 Single Locus Complementary Sex Determination in Hymenoptera: An ‘Unintelligent’ Design? Front. Zool. 3: 1 10.1186/1742-9994-3-1 16393347 PMC1360072

[bib94] Zimin A. V., Marçais G., Puiu D., Roberts M., Salzberg S. L., 2013 The MaSuRCA Genome Assembler. Bioinformatics 29: 2669–2677. 10.1093/bioinformatics/btt476 23990416 PMC3799473

